# Aortic root movement correlation with the function of the left ventricle

**DOI:** 10.1038/s41598-021-83278-x

**Published:** 2021-02-24

**Authors:** Piotr Karwat, Ziemowit Klimonda, Grzegorz Styczyński, Cezary Szmigielski, Jerzy Litniewski

**Affiliations:** 1 Institute of Fundamental Technological Research, Polish Academy of Sciences, Pawińskiego 5B, 02-106 Warsaw, Poland; 2 Department of Internal Medicine, Hypertension and Angiology, Medical University of Warsaw, Banacha 1A, 02-097 Warsaw, Poland

**Keywords:** Cardiology, Echocardiography

## Abstract

Echocardiographic assessment of systolic and diastolic function of the heart is often limited by image quality. However, the aortic root is well visualized in most patients. We hypothesize that the aortic root motion may correlate with the systolic and diastolic function of the left ventricle of the heart. Data obtained from 101 healthy volunteers (mean age 46.6 ± 12.4) was used in the study. The data contained sequences of standard two-dimensional (2D) echocardiographic B-mode (brightness mode, classical ultrasound grayscale presentation) images corresponding to single cardiac cycles. They also included sets of standard echocardiographic Doppler parameters of the left ventricular systolic and diastolic function. For each B-mode image sequence, the aortic root was tracked with use of a correlation tracking algorithm and systolic and diastolic values of traveled distances and velocities were determined. The aortic root motion parameters were correlated with the standard Doppler parameters used for the assessment of LV function. The aortic root diastolic distance (ARDD) mean value was 1.66 ± 0.26 cm and showed significant, moderate correlation (r up to 0.59, *p* < 0.0001) with selected left ventricular diastolic Doppler parameters. The aortic root maximal diastolic velocity (ARDV) was 10.8 ± 2.4 cm/s and also correlated (r up to 0.51, *p* < 0.0001) with some left ventricular diastolic Doppler parameters. The aortic root systolic distance (ARSD) was 1.63 ± 0.19 cm and showed no significant moderate correlation (all r values < 0.40). The aortic root maximal systolic velocity (ARSV) was 9.2 ± 1.6 cm/s and correlated in moderate range only with peak systolic velocity of medial mitral annulus (r = 0.44, *p* < 0.0001). Based on these results, we conclude, that in healthy subjects, aortic root motion parameters correlate significantly with established measurements of left ventricular function. Aortic root motion parameters can be especially useful in patients with low ultrasound image quality precluding usage of typical LV function parameters.

## Introduction

Echocardiography is the most frequently used imaging modality worldwide^1^. Unfortunately, in real-life clinical environment, many echocardiographic studies have low signal to noise ratio and low image quality that limits significantly the assessment of systolic and diastolic function of the heart, especially from the apical views.

However, there may be unconventional approaches to the heart assessment that can be determined from the aortic root movement. The aorta is a geometrically complex organ that begins at the bulb-shaped root^2^. The aortic root is attached at the top of the heart and is directly connected to the cardiac fibrous skeleton. This large structure is well visualized in most patients, even in those with apparently low image quality. It is often thoroughly examined for aortic valve and aortic root abnormalities, but not used for the assessment of the whole LV function. Therefore, due to the ease with which it can be visualized, and its high clinical importance, aortic root movement has a potential to be used as an echocardiographic marker of the LV function, after additional image analysis.

An accurate detection, delineation, and tracking of aortic root in dynamic cardiac imaging is a complex task. However, researching that dynamic aspects of aortic movement is important for the assessment of cardiovascular system^3^. In cardiovascular imaging, the movement of the heart structures is the base for cardiac functional imaging. Dynamic assessment of the movement in situ of the cardiac structures is only recently possible due to modern imaging techniques. Several different imaging techniques have been used in aortic movement analysis, especially cardiac magnetic resonance (CMR) and cardiac computed tomography (CCT) with their intrinsic advantages and disadvantages. However, echocardiography has the widest applicability and, what is especially important for functional analysis, it has uniquely high time resolution, crucial for reliable movement assessment^4^.

The study involved the acquisition and analysis of echocardiographic data to characterize the aortic root motion and to assess its relationship with the left ventricular systolic and diastolic cardiac parameters. The aortic root was tracked within a standard two dimensional (2D) B-mode (brightness mode, classical ultrasound grayscale presentation) image sequence corresponding to a single cardiac cycle. Then several parameters characterizing the aortic root motion were determined. In the next step the aortic root motion parameters were subjected to statistical analysis including their correlations with the standard echocardiographic Doppler spectral flow and Doppler tissue imaging parameters.

In this study, we hypothesize that the aortic root movement recorded with echocardiographic parasternal long axis (PLAX) view may correlate with diastolic and systolic function of the left ventricle of the heart.

## Methods

### Data acquisition

This study was conducted retrospectively from data collected in accordance with the ethical standards of the institutional and national research committee and with the 1964 Helsinki Declaration and its later amendments or comparable ethical standards. The protocol was approved by Bioethics Committee of Medical University of Warsaw. Informed consent was obtained from all individual participants included in the study.

The data were collected in a tertiary care university hospital. A group of 101 healthy volunteers participated in the study. It included 45 men and 56 women with a mean age (± standard deviation) of 46.6 ± 12.4 years. More detailed characteristics of the participants are shown in Table 1.

**Table 1:** Basic information on the participants.

	Median	Mean	SD
Age [yr]	47	47	12
Body weight [kg]	71	74	14
Body height [cm]	172	171	8
Body surface area [m^2^]	1.9	1.9	0.2
Systolic blood pressure [mmHg]	122	123	11
Diastolic blood pressure [mmHg]	76	77	7
Pulse pressure [mmHg]	45	46	8
Heart rate [s^−1^]	65	65	9

**Figure 1:**
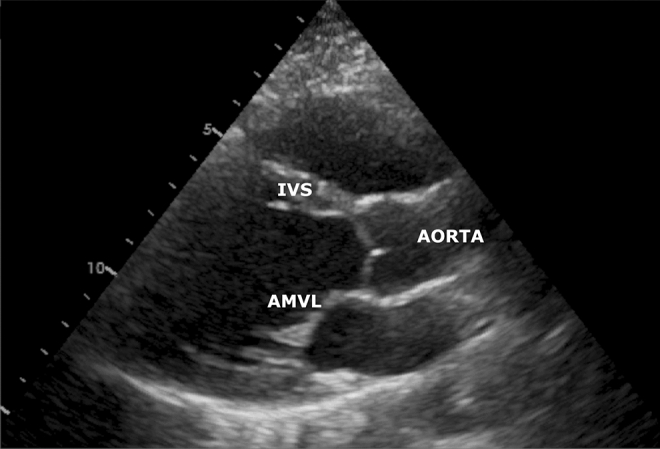
B-mode image of the heart in the PLAX view. Additionally marked structures are: the beginning of the aorta, the anterior mitral valve leaflet (AMVL), and the interventricular septum (IVS).

The 2D studies were recorded according to standard imaging guidelines^5^. The data were acquired with the Vivid E9 scanner, (GE Vingmed Ultrasound AS, Horten, Norway) with the M5S-D (1.7–3.3 MHz) cardiac probe. The image acquisition was synchronized with the ECG signal. The data of each participant contained a set of standard echocardiographic 2D images and Doppler parameters. An ultrasound B-mode image sequence corresponding to a single cardiac cycle in a standard parasternal long axis (PLAX) view was used for the aortic movement tracking (example shown in Fig. 1).

All the further data processing, including aortic root tracking and statistical analysis, was done in Matlab 2018a (MathWorks, Natick, Massachusetts, USA).

### Aortic root tracking

The aortic root is usually a strong source of ultrasonic echoes and is usually very clearly visible in B-mode PLAX images, even in patients with otherwise low ultrasound image quality. It is also a well delineated structure, which maintains its shape and orientation in the PLAX view throughout the entire cardiac cycle. Therefore, it can be successfully tracked using a simple correlation tracking algorithm. There are, however, a few issues that can be encountered, when using this approach.

First, the aortic root is adjacent to heart structures whose movement interferes with the tracking. These structures include the rapidly moving aortic valve leaflets and anterior mitral valve leaflet (AMVL), or the interventricular septum (IVS), being subject to shape deformations during cardiac cycle. To reduce their influence on the tracking algorithm performance, it is necessary to properly define the region of interest (ROI) that will be tracked during analysis. In our work, the ROI was selected manually—it was not a purpose of this work to develop a fully autonomous method. The selection included the aortic root with a narrow margin, to minimize the influence of the IVS and AMVL movement.

Furthermore, finite pixel density of the B-mode data leads to discretization errors and limits sensitivity of the tracking method. To reduce these problems we chose to upsample the B-mode images. The signal also underwent smoothing filtration to reduce fluctuations of the speckle pattern, which in turn limited the variance of the displacement estimation. Finally, the signal was thresholded to remove low-amplitude image areas, unrelated to the walls of the aortic root. The above operations were performed as signal preprocessing in the tracking algorithm.

**Figure 2:**
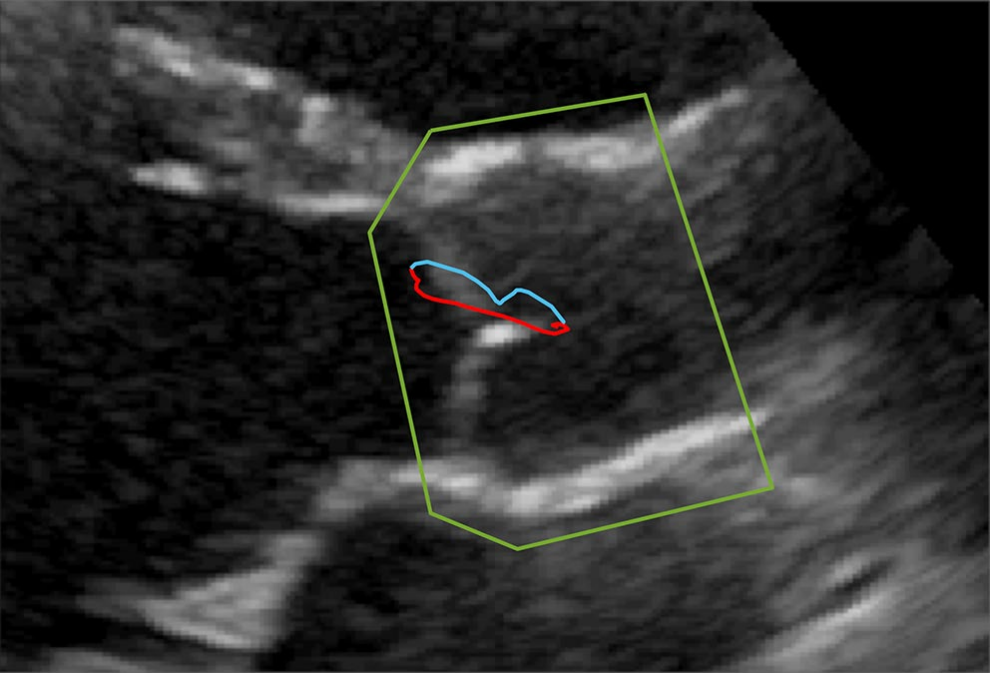
Close-up of the aortic root with its systolic and diastolic motion paths marked with a red and blue lines, respectively, and the region of interest (ROI) marked with a green line.

After the preprocessing, the tracking algorithm performs a 2-dimensional cross-correlation of two consecutive frames of a B-mode picture, frame no. 1 (limited to ROI) and frame no. 2 that follows. The displacement value, at which the cross-correlation function reaches its maximum, is the aortic root displacement between the 1st and 2nd frame. Next, the position of the ROI is moved, using the calculated displacement, so that it covers the aortic root in the 2nd frame. Then, the displacement is calculated as before, but for the next pair of frames (2nd and 3rd frames). The procedure repeats until all pairs of subsequent frames are processed. Then, the displacement sequence is accumulated to obtain the aortic root motion path for the entire cardiac cycle (example shown in Fig. 2 and Supplementary Video S1).

### Aortic root motion parameters

Based on the aortic root position as a function of time, a number of parameters characterizing its movement were determined, including distances traveled by the aortic root, and its maximal velocities. These are: aortic root systolic distance (ARSD), aortic root diastolic distance (ARDD), aortic root systolic velocity (ARSV), and aortic root diastolic velocity (ARDV).

It is worth emphasizing that the distances: ARSD and ARDD, are not measured in a straight line, but along the complex path of the aortic root motion. Therefore, the systolic and diastolic distances ARSD and ARDD are usually not equal.

It must also be noted that the aortic root velocities, being the derivatives of the aortic root positions, are characterized by high variance. To reduce it, the velocities were low-pass filtered using short triangular window (filter coefficients were: [0.25; 0.50; 0.25]). This, however, leads to underestimation of the maximum velocities which are effectively determined based on a time window of increased length. Therefore, when comparing the values of the maximum velocities obtained in different studies, one should remember about the influence of the time window.

### Statistical analysis

The aortic root motion parameters of 101 participants were statistically analyzed in several ways. Their distributions were tested for normality using Shapiro–Wilk test and a confidence level of 0.95 (significance level of 0.05) was used in analysis for significance cut off values.

Each of the aortic root motion parameters was cross-correlated with each of the standard echocardiographic Doppler parameters of the left ventricle, typically used in standard cardiac diagnostics. The Spearman’s rank correlation coefficient r and the p-value were calculated. Strength of relationship was defined as: |r|≥ 0.80, very strong; |r|= 0.60–0.79, strong; |r|= 0.40–0.59, moderate; |r|= 0.20–0.39, weak, |r|= 0.00–0.19, negligible.

## Results

**Figure 3:**
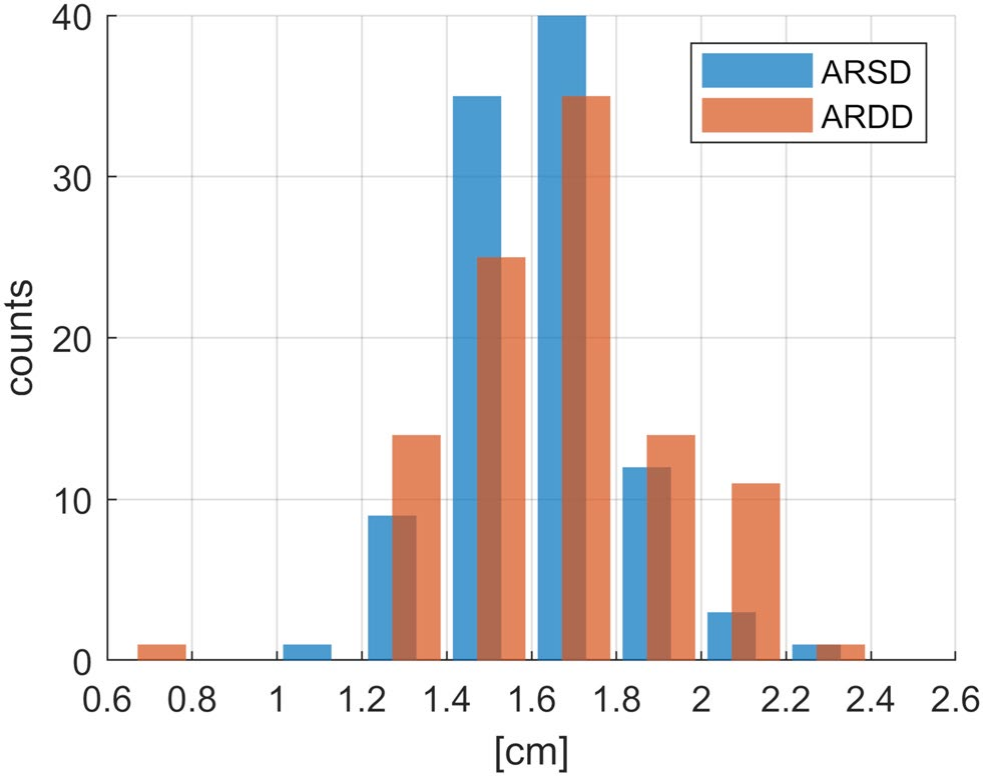
Distributions of the aortic root distances.

**Figure 4:**
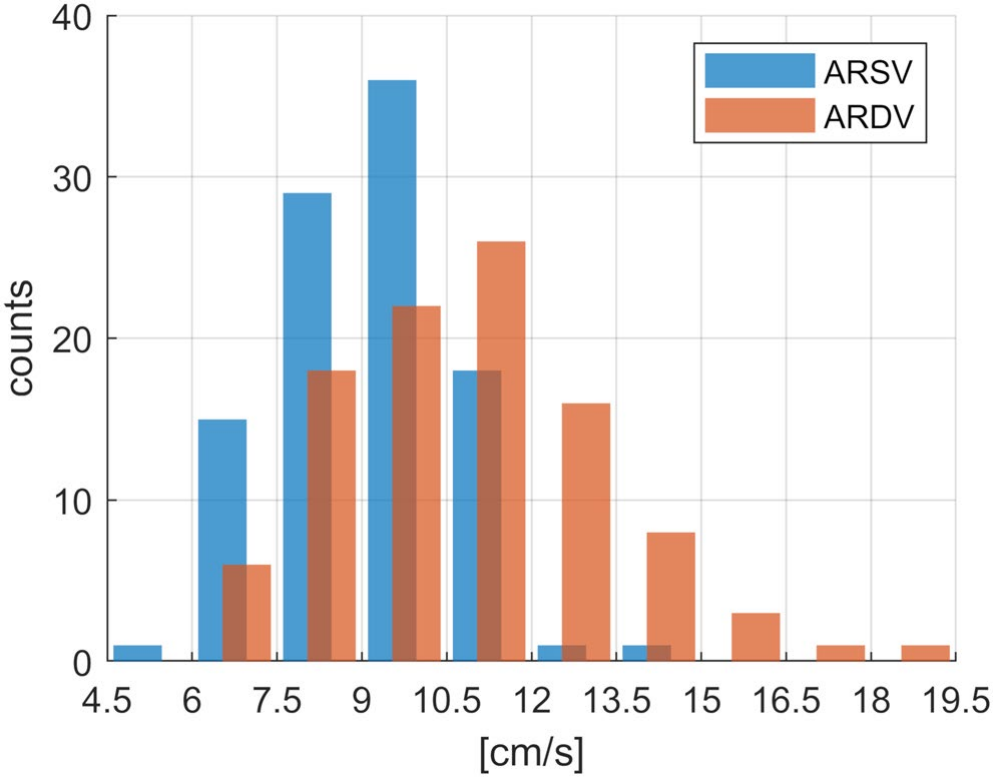
Distributions of the aortic root velocities.

The tracked aortic root motion paths and the related aortic root motion parameters, as well as the established Doppler parameters, are included in Supplementary Data S2. The distributions of the four studied aortic root motion parameters are shown in Figs. 3 and 4. Basic statistics of both the aortic root motion parameters and the established Doppler parameters are shown in Table 2. According to the results of the Shapiro–Wilk test, all the distributions of the aortic root motion parameters are normal. However, this is not the case for most of the Doppler parameters. Therefore, we decided to use Spearman’s rank correlation coefficient. The correlations between aortic root parameters and standard echocardiographic spectral and tissue Doppler parameters are shown in Table 3.

**Table 2:** Basic statistics of the aortic root parameters and standard Doppler parameters.

	Median	Mean	SD	Skewness	Normality
**Aortic root motion parameters**					
ARSD [cm]	1.62	1.63	0.19	0.53	Yes
ARDD [cm]	1.66	1.66	0.26	0.01	Yes
ARSV [cm/s]	9.28	9.22	1.55	0.23	Yes
ARDV [cm/s]	10.7	10.8	2.4	0.52	Yes
**Standard Doppler parameters**					
SV	77	78	16	0.38	No
CO	5.1	5.3	1.3	0.83	No
s' med	0.080	0.084	0.014	0.22	No
E	0.82	0.81	0.14	− 0.36	Yes
A	0.61	0.63	0.14	0.42	Yes
E/A	1.4	1.4	0.4	1.02	No
e`lat	0.14	0.14	0.03	0.30	Yes
e`med	0.10	0.10	0.02	0.29	No
e` mean	0.12	0.12	0.03	0.27	Yes
E/e`	6.9	7.0	1.6	1.02	No

*ARSD* aortic root systolic distance, *ARDD* aortic root diastolic distance, *ARSV* aortic root maximal systolic velocity, *ARDV* aortic root maximal diastolic velocity, *SV* stroke volume, *CO* cardiac output, *s′ med* peak systolic velocity of medial mitral annulus by tissue Doppler, *E* transmitral E wave velocity, *A* transmitral A wave velocity, *E/A* ratio of the transmitral E wave velocity and A wave velocity, *e′ lat* tissue Doppler lateral e′ wave velocity, *e′ med* tissue Doppler medial e′ wave velocity, *e′ mean* tissue Doppler mean e′ wave velocity, *E/e*′ ratio of transmitral E wave velocity to mean tissue Doppler e′ wave velocity.

**Table 3:** Correlations between aortic root parameters and standard echocardiographic Doppler parameters.

	r (*p* value)			
	ARSD	ARDD	ARSV	ARDV
SV	+ 0.32 (< 0.01)^*#*^	+ 0.11 (0.28)	+ 0.14 (0.17)	− 0.04 (0.71)
CO	+ 0.31 (< 0.01)^*#*^	+ 0.02 (0.82)	+ 0.28 (< 0.01)^*#*^	+ 0.05 (0.65)
s′ med	+ 0.33 (< 0.001)^*#*^	+ 0.22 (0.03)^*#*^	+ 0.44 (< 0.0001) *	+ 0.28 (< 0.01)^*#*^
E	+ 0.08 (0.45)	+ 0.31 (< 0.01)^*#*^	+ 0.14 (0.18)	+ 0.22 (0.03)^*#*^
A	+ 0.06 (0.58)	− 0.38 (< 0.0001)^*#*^	+ 0.07 (0.49)	− 0.19 (0.06)
E/A	+ 0.01 (0.92)	+ 0.55 (< 0.0001) *	+ 0.01 (0.94)	+ 0.33 (< 0.01)^*#*^
e`lat	+ 0.13 (0.20)	+ 0.52 (< 0.0001) *	+ 0.23 (0.02)^*#*^	+ 0.46 (< 0.0001) *
e`med	+ 0.16 (0.11)	+ 0.59 (< 0.0001) *	+ 0.24 (0.02)^*#*^	+ 0.51 (< 0.0001) *
e` mean	+ 0.15 (0.14)	+ 0.58 (< 0.0001) *	+ 0.24 (0.02)^*#*^	+ 0.51 (< 0.0001) *
E/e`	− 0.15 (0.14)	− 0.39 (< 0.0001)^*#*^	− 0.17 (0.09)	− 0.38 (< 0.0001)^*#*^

*Moderate correlation.

^#^Weak or negligible correlation.

The aortic root systolic parameters (ARSD and ARSV) correlated generally weakly (only in one case—moderately) with systolic Doppler parameters, like stroke volume (SV), cardiac output (CO), and s′ medial. The aortic root diastolic parameters (ARDD and ARDV) correlated moderately with left ventricular diastolic parameters such as: e′ mean, e′ medial, e′ lateral, E/A, and E/e’. The ARDD correlated slightly better than ARDV.

## Discussion

The results of our study showed that the aortic root diastolic distance (ARDD) significantly correlated with the established and widely used left ventricular diastolic parameters, such as E/A, E/e’, e’. However, there were no significant correlations with several selected systolic parameters recorded with Doppler flow and Doppler tissue imaging (DTI), like stroke volume (SV), cardiac output (CO), and s′ medial. Additionally, another new parameter of the aortic movement, the aortic root maximal diastolic velocity (ARDV), showed weaker correlations than diastolic distance. Researching these dynamic aspects of aortic movement is especially important for the assessment of the cardiovascular system^3^.

Normally, in heart cycle, during systole, the aortic annulus is pulled along the left ventricle in systole and retracted in diastole. The movement of the aortic annulus towards the apex results in longitudinal stretch in the ascending aorta. Accordingly, the major systolic movement of the aortic root is downward motion. The in-plane motion and clockwise axial twist were found to be not significant for the longitudinal deformation^6^. What is especially important, the aortic root displacement is reduced in patients with the left ventricular dysfunction. Therefore, it can be especially useful in large population of patients assessed for LV function^7^. On the other hand, in some studies, it was elevated in aortic valve regurgitation due to ventricular compensation and increased stroke volume^7^. However, our approach looks especially promising for diastolic assessment, which is often even more difficult than systolic assessment. An assessment of the left ventricular diastolic function is an integral part of every comprehensive echocardiographic study^8^.

In our study, ARDD showed a positive correlation with E velocity and negative with A velocity. Among cardiac parameters, mitral peak E-wave velocity, E/A ratio, E/e′ ratio, relate best with early occurring LV diastolic pressures. The mitral E-wave reflects primarily the LA-LV pressure gradient during early diastole and is affected by preload, and LV relaxation^9^. The rapid pressure drop and the relaxation of the LV give the suction effect that pulls blood into the LV. Additionally, in situ, at the beginning of diastole, in normal conditions, an aortic recoil facilitates LV lengthening and early diastolic filling. This leads to higher E velocity and improved LV early feeling. However, A wave correlates with LV end-diastolic pressure. The mitral A-wave velocity reflects the LA-LV pressure gradient during late diastole, which is affected by LV compliance and LA contractile function. An aortic recoil potentially might also influence impedance to atrial emptying, decrease atrial pressure, and facilitate LA-LV pressure gradient in normal hearts. However, with increasing aortic stiffness, any beneficial contribution of aortic stretch and recoil to early diastolic filling may disappear^10^. The LV diastolic dysfunction is very common in patients, and it is usually the result of impaired LV relaxation with or without reduced restoring myocardial forces. Early diastolic suction and increased LV chamber stiffness can also increase cardiac filling pressures. In clinical scenario of cardiac abnormalities, the diastolic abnormalities are usually earlier than systolic abnormalities. Impaired aortic elasticity, due to aging, hypertension or diabetes, is also believed to play some independent role in the pathogenesis of LV dysfunction. Therefore, additionally to loading conditions, passive cardiac and vascular tissue properties and myocardial relaxation are affecting tissue deformation. Also, the motion of any specific part of the myocardium is influenced by overall motion (translational effects) and tethering of the heart regions. Hence, any measure of cardiac function should be interpreted carefully in the context of loading condition of the heart, wall thickness, and the shape of the ventricle.

Previously, in echocardiography, the aortic movement was mostly studied with the M-mode (motion mode, image of a single scan line over time) imaging, that is gradually disappearing from most echocardiographic guidelines^1^. In M-mode, the aortic walls are moving anterior in systole and posterior in diastole. One study, also based on M-mode, assessed early diastolic motion of the posterior aortic root and used the slope of early diastolic posterior aortic root motion^11^. However, this was limited to one direction technique, therefore providing few data about the whole aortic root. In another recent publication, the systolic movement of aorta was described as downward, anterior and lateral^12^. Throughout the heart cycle, it parallels the motion of the mitral annulus in the longitudinal axis of the left ventricle^13^. Early studies with M-mode postulated that systolic aortic root motion is a response to the action of the whole LV. Some authors believed that it should be attributed mainly to LV systolic function^12^. On the other hand, we found significant correlations between diastolic aortic root motion and diastolic LV parameters.

Our work adds data about the aorta complex path of motion, both in systole and in diastole, and its parameters related to the LV function. Therefore, the approach showed in our study provides potentially important information about the movement of the aortic root in healthy subjects, with currently the most often used 2D echocardiography^14^. When interpreting the results, it is important to keep in mind, that in our study we provide information about subjects with normal hearts, therefore, without wide variability in various factors influenced by cardiovascular diseases^15^.

We studied group of patients without significant risk of increased aortic stiffness, that can form additional load on the left ventricle^16^. Some authors proposed that alterations in both LV and aortic physiology may play a role in predisposition to heart failure, including heart failure with preserved ejection fraction (HFpEF)^17^. They showed that the aortic stiffening was related to global longitudinal function of the LV^17^. Also, impaired LV diastolic function and increased aortic stiffness have been postulated^18^. Diastolic recoil of the aorta, which is stretched during systole, can facilitate LV filling and ejection^19^.

In the future, non-invasive aortic root movement measures may identify patients at higher risk for progressive aortic enlargement and adverse clinical outcomes, potentially allowing for closer monitoring and more appropriate therapy in patients with cardiovascular diseases^6,7,20^. This is especially important in populations with high risk of aortic aneurysm and aortic dissection, including patients with bicuspid aortic valve (BAV) and collagen diseases, especially Marfan syndrome. Unfortunately, the aortic diameter is not a good predictor of aortic dissection and additional parameters should be studied to decrease morbidity and mortality in those patients^21^. Longobardo L et al. showed that impairment of elastic properties of the ascending aorta is a predictor of aortic complications in patients with bicuspid aortic valve^22,23^. In these studies the authors stated that the alterations of functionality often precede the anatomical changes in cardiovascular pathophysiology. Guala et al. found that the proximal aorta longitudinal strain, but not circumferential strain and distensibility, was an independent predictor of the aortic root diameter growth rate in cardiac magnetic resonance (CMR) of Marfan syndrome patients^24^. The axial motion of aortic root due to ventricular traction was suggested to contribute to aortic dissection by increasing its longitudinal stress. The largest aortic wall stress increase due to aortic root displacement was found approximately 20 mm above the sino-tubular junction, and that is often the place of aortic dissection tear^7^. However, neither axial nor in-plane motion could directly lead to aortic dissection, therefore more attributes related to aortic structure, motion and hemodynamics of aortic flow should be evaluated^6^.

We conclude that in healthy subjects, the aortic root motion parameters correlate significantly with established measurements of the left ventricular function. Aortic root motion parameters can be useful in patients with low ultrasound image quality, especially when there are no apical views available for routine left ventricular function assessment. New quantifiable imaging parameters that can be universally applied, like ARDD and ARDV, could be a further advance towards improving cardiovascular prevention, monitoring and therapy. In the future, to have complete information about the cardiac conditions, the aortic root motion may be included in the comprehensive analysis of the heart.

### Limitations of the study

Nonetheless, our findings must be interpreted with caution and several limitations should be borne in mind. The first is the moderate number of participants included in the study and their lack of cardiovascular diseases, as healthy subjects. This might result in the obtained correlation values being different from values for the population with cardiovascular conditions. In the future, we plan to increase the number of participants by including subjects with systolic and diastolic dysfunction.

The second limitation is due to the use of the 2D images for the motion tracking. It has to be kept in mind that the aortic root motion parameters presented in this paper do not include the motion component that is perpendicular to the PLAX plane. In quest to include any movement direction, one can consider additionally using of the aortic root motion path obtained from parasternal short axis (PSAX) view, where the aortic root is clearly visible as well. The PSAX plane is perpendicular to the PLAX plane, and can therefore provide some additional information. But this approach would require an effective method for merging of two 2D motion paths obtained for different cardiac cycles into a 3D motion path, and therefore generate further technical challenges. The 3D motion information can also be obtained by tracking the aortic root directly on volumetric data available in 3D echocardiography, especially in 3D fusion echocardiography^25,26^.

## Supplementary Information

Supplementary Video S1.

Supplementary Data S2.

## Data Availability

The ultrasound B-mode image sequences are available from the corresponding author on reasonable request. The tracked aortic root motion paths and the related aortic root motion parameters, as well as the established Doppler parameters, are included in the Supplementary Information files (Supplementary Data S2).
